# Predictive Maintenance: An Autoencoder Anomaly-Based Approach for a 3 DoF Delta Robot

**DOI:** 10.3390/s21216979

**Published:** 2021-10-21

**Authors:** Kiavash Fathi, Hans Wernher van de Venn, Marcel Honegger

**Affiliations:** Institute of Mechatronic Systems, Zurich University of Applied Sciences, 8400 Winterthur, Switzerland; fath@zhaw.ch (K.F.); honr@zhaw.ch (M.H.)

**Keywords:** predictive maintenance, anomaly detection, autoencoder, gaussian processes, deep learning, data-driven maintenance

## Abstract

Performing predictive maintenance (PdM) is challenging for many reasons. Dealing with large datasets which may not contain run-to-failure data (R2F) complicates PdM even more. When no R2F data are available, identifying condition indicators (CIs), estimating the health index (HI), and thereafter, calculating a degradation model for predicting the remaining useful lifetime (RUL) are merely impossible using supervised learning. In this paper, a 3 DoF delta robot used for pick and place task is studied. In the proposed method, autoencoders (AEs) are used to predict when maintenance is required based on the signal sequence distribution and anomaly detection, which is vital when no R2F data are available. Due to the sequential nature of the data, nonlinearity of the system, and correlations between parameter time-series, convolutional layers are used for feature extraction. Thereafter, a sigmoid function is used to predict the probability of having an anomaly given CIs acquired from AEs. This function can be manually tuned given the sensitivity of the system or optimized by solving a minimax problem. Moreover, the proposed architecture can be used for fault localization for the specified system. Additionally, the proposed method can calculate RUL using Gaussian process (GP), as a degradation model, given HI values as its input.

## 1. Introduction

There are numerous methods for maintaining systems in industry, namely corrective maintenance, preventive maintenance and predictive maintenance. Corrective maintenance is performed once a failure has occurred in the system. Accordingly, the defected system is repaired either immediately or later [1]. The preventive maintenance; however, is executed prior to a system failure. Moreover, preventive maintenance is further divided into two categories: pre-scheduled and condition-based maintenance. In pre-scheduled maintenance, given the historical data from the machines, average historic lifetime is acquired, and maintenance is executed accordingly. On the other hand, condition-based maintenance takes the current status of the system into account for evaluating whether the system requires any maintenance [2]. The aforementioned methods result in an inefficient scheduling of maintenance, given the fact that no future estimations of the system’s condition are used to make more informative decisions [3]. Furthermore, there are four main approaches for performing PdM [4,5]: reliability statistics method, physical model-based method, knowledge-based method and data-driven method. In the first method, information required for performing maintenance is encapsulated in different probability density functions [6,7]. Examples of this approach include Bayesian method and fuzzy logic. This method relies on the extracted statistical characteristics of gathered data for fault prediction and thus, no mathematical model is required. On the contrary, the physical model-based approach, requires the mathematical model of the system, so that the performance degradation of the system can be inferred [8,9]. This method requires a lot of support from experts and normally, the degradation models are too complicated to be calculated given the fact that degradation mechanisms are simplified in the model derivation procedure. In addition, knowledge-based systems try to integrate conventional failure techniques with heuristics acquired from expert experience to perform PdM. Similar to physical model-based method, such an approach requires extensive support from an expert, which is not practical when the studied system is complex. Additionally, similar failure behavior is required to be used as a reference for inferring anomaly in the system, which is not always available. Lastly, the data-driven approaches merely require data from the studied system and no further mathematical modelling, or probabilistic inference is required [10,11]. This approach significantly increases productivity when integrating domain knowledge from the studied system into the model is hard, or impossible due to the complexity of the system. By analyzing the current literature one can see that the current methods for implementing PdM either require a complete R2F data or require an analytical model. As has been formerly pointed out, it can be hard and time consuming to acquire a mathematical model which is accurate enough for PdM when the studied system is relatively complex. On the other hand, it is not always possible to have access to a R2F dataset or it is not available, either due to the high costs or the time required for completing the R2F dataset. These two problems extremely hinder the use R2F-based PdM methods in industry. Additionally, as the system becomes more complex, it is not possible to acquire R2F data from all the possible failure scenarios. The aforementioned issues can prevent the optimal development and use of PdM algorithms. In this paper, we have developed an architecture which solves the issue with availability of R2F data as it will be explained shortly. In what follows, more in-depth details about PdM are given. PdM is an optimal approach for performing condition-based maintenance of systems, based on real-time monitoring and identifying CIs which result in a failure when deteriorated [12]. In fact, using CIs, it is possible to detect fault or degradation, when any of the CIs depicts a symptom of a near future malfunctioning of the system. Moreover, different steps of PdM include fault detection, fault localization, and afterwards estimating the RUL of the studied system [13,14]. In comparison with other classical methods such as corrective and preventive maintenance, such an approach can significantly reduce the maintenance costs, since almost the entire useful life of the system can be used. The chosen approach in this paper for performing PdM is data-driven maintenance planning framework [15]. As discussed above, data-driven models have proven to be effective when the complexity of the studied system prevents using model-based approaches [16]. In fact, data-driven models tend to reveal the correlations between collected sensor data and thus can calculate corresponding system formation such as RUL [17]. Moreover, given the improvements in data acquisition and availability of data, the aforementioned data-driven PdM has even caught much attention lately [18,19]. It is worth noting that the data-driven models are divided into two groups. The first group tries to find mappings from multi-sensory data to the RUL. The second group however, as the proposed method, tries to transform the available high-dimensional data into a one-dimensional HI and calculate the RUL based on this new value. Moreover, the second group has shown better generalization and higher precision on the available public datasets [17]. Additionally, the possibility of gathering data from multiple sensors in Industry 4.0 environments, has also led to the increased use of PdM [20]. It can be said that once enough data from all parts of the studied process can be gathered, PdM can be employed. As a result, maintenance costs and downtime will be reduced, and the overall productivity will increase in an Industry 4.0 setting [21]. Nonetheless, one important obstacle is normally neglected in Industry 4.0 and Digital twin cases. Usually, no historical R2F dataset is available in industry [22] and it is very unlikely that one will find examples in the relevant literature that fit a current problem in detail. In the conducted study, the main focus is to perform PdM without having R2F data from the studied system. Such an approach is missing from the current literature, and thus the proposed method can be of great importance when gathering such data is not feasible. Furthermore, one of the main challenges of data-driven PdM is tackling with large, high-dimensional datasets. Additionally, identifying CIs and extracting informative features from the available data can be tedious, when manual feature engineering is deployed. For addressing the above-mentioned problems, machine learning and deep-learning approaches can be used [23,24,25]. There has been an extensive study on different machine learning and deep-learning structures such as support vector machines, convolutional neural networks, long short-term memory (LSTM) and AEs in the domain of PdM [26,27,28,29,30]. Consequently, the problem with traditional machine learning approaches such as support vector machines is that these methods do not capture temporal dependencies in the available data and neglect anomalies that are time dependent. Moreover, features derived from deep-learning models tend to preserve useful information in the original data compared to the output of feature engineering required in machine learning models [31]. Therefore, for attaining better results from trained models, the extensive use of deep neural networks is vital [32]. However, it is worth noting that a deep feed-forward neural network will not be effective either, given that this structure requires temporal dependencies in advance [33]. Furthermore, LSTM models are not sufficiently accurate when the output dimension is large [34,35]. On the other hand, convolutional neural networks have been proven to be effective for extracting features from raw data. Additionally, this structure was designed for high-dimensional data of images. This characteristic of this type of neural network makes it perfect for feature extraction in PdM even with high-dimensional data [36]. Likewise, AEs have been successfully implemented for feature extraction, dimensionality reduction, anomaly detection and time-series prediction [37,38,39]. Given the performance of AEs and convolutional neural networks in handling high-dimensional data and extracting features, it has been decided to use these architectures in the proposed method. Additionally, what is generally missing from conducted studies is a hybrid architecture that benefits from different capabilities of the available data-driven models for anomaly detection and using this information for calculating RUL given that no R2F data are available. In fact, to the best of our knowledge, there are currently no models which try to deal with PdM without R2F data in an optimized way as proposed in this paper. The previous studies targeting PdM, normally consider a complete data set of R2F data [37]. Similarly, some other are only limited to feature extraction and anomaly detection of their system in hand [40]. In the proposed method, an AE with convolutional layers is trained to find CIs and calculate the corresponding HI using a minimax optimized sigmoid function, and finally determine when maintenance is required using a GP as a degradation model. Essentially, the proposed method tries to cluster data based on the distribution of signal sequences of different tasks. The aforementioned signal sequences are gathered from a 3 DoF delta robot, used for picking and placing parts in a smart factory. This architecture combines the semi-supervised training and feature extracting of AEs with the power of convolutional neural network for finding spatial information in the available signal sequences. Furthermore, reconstruction error in the proposed method is used to come up with a CI which is then passed to a minimax optimized sigmoid function to calculate the HI. Additionally, HI is interpreted as the probability of the current system failing given the formerly mentioned CI. The HI can also be interpreted as an anomaly probability value where by HI=0, the system is operating as expected [41]. It is worth noting that the proposed HI is the complement of HI introduced in some literature [17]. After acquiring the HI, a.k.a. anomaly probability values, a GP as a degradation model is trained to predict the time-series of HI values. Based on the predicted values, it is possible to calculate the RUL of the studied system given different previously chosen thresholds [22,42]. In short, the main contributions of the proposed method are the following:

1. Given the semi-supervised nature of the method, there is no need for R2F data for training the model, which is vital when gathering such data can be dangerous or economically infeasible.
2. The proposed method does not require hand designed features for training the models, which makes the training stage easier when no or too little domain knowledge about the system is available or when a large, high-dimensional dataset must be tackled.
3. The proposed method can also be used to classify the task and determine which task is going to fail, based on the similarity of distribution of the signal sequence.
4. By having the output of the proposed method, a probability can be assigned, which describes how probable it is that the system is going to fail. This probability includes a slack variable which determines how much deviation from reference values is allowed. Additionally, it is also possible to determine the sensitivity and rate of changes of the proposed method to deviation from the reference values or find optimal values by solving a minimax problem.
5. Given the studied system, the proposed method is capable of pinpointing where the problem originates from, regardless of the number of motors misbehaving. Moreover, the output of fault localization shows that the trained model has learned some correlations between different parameters of the studied system.
6. The trained GP does not require numerous data points to predict the anomaly values. Moreover, the dedicated GP for regression does not require a long time to be (re)trained. These characteristics make GP ideal for online prediction of anomaly values.

In essence, the hypothesis is that by with enough data points from the delta robot’s error-free operating condition, it is possible to train a deep neural network, capable of determining the distribution of error-free signal sequences of the studied system. Afterwards, based on the deviation from the determined distributions, it is possible to calculate a new CI. This new CI is then transformed into HI which describes the probability of having an anomaly. Afterwards, this anomaly value is predicted using GP as a degradation model. This prediction of this value can be used for calculating RUL of the studied system based on some given thresholds, without having access to any R2F data.

## 2. Material and Methods

In this section, different deep-learning architectures used in the proposed method along with the dynamics and parameters of the delta robot are introduced.

### 2.1. Autoencoder (AE)

AEs try to replicate their input at their output through an internal representation. The aforementioned internal representation is in fact a bottleneck in a higher or lower-dimensional space from which the decoded output is calculated. The only constraint is to have a sufficiently complex space which can learn the distribution of the provided data. AEs are made of the following parts:

- encoder *f*
- internal representation *z*
- decoder *g*

Given an input vector *x*, the encoder part of AE, which can be a single or a multi-layer structure, finds an internal representation of the provided input. The internal representation is in fact the latent space, in a higher or lower-dimensional space, capable of representing the provided input [43]. Therefore, it can be claimed that

(1) z=f(x)

Afterwards, by having the internal representation, the decoder part of the AE, tries to estimate the formerly given input, denoted as $\hat{x}$, based on given *z*. The aforementioned can be formulated as

(2) $$\hat{x}=g\left(z\right)=g\left(f\left(x\right)\right)$$

The reconstruction error is the difference between the input and the output of the AE [44]. In the conducted study, the loss function of the neural network is the mean squared error (MSE). Generality of the trained model, plays a significant role in the performance of the proposed method. By having AEs which merely copy the input at their output, no insight is provided whether a signal sequence is error-free or if maintenance is required. In respect to AEs, for increasing the generality of the trained model, it has been decided to use deep AEs [45]. Additionally, using convolutional layers, instead of having deep dense neural networks, locality of the features are also taken into account for finding the underlying distribution of error-free signal sequence.

In this paper, the PdM of a delta robot without any R2F data is studied. Consequently, given that no R2F data are available, supervised models cannot be used for training a PdM algorithm. Therefore, the ideal architecture for the proposed method is AE for the following reasons:

- AE does not require any labels as the input is the output of the neural network.
- AE can capture the underlying error-free signal sequence distribution.
- Data shifts from the learned distributions can easily be acquired from the reconstruction error at the output of the AE.

Moreover, the delta robot is used to perform pick and place of barrels and springs in a pen production line. The barrel and spring of a pen are sent to the station where the delta robot is located, with a tray containing different parts of the customized pen. More details about the delta robot are provided in Section 2.3. In the proposed method, two datasets from barrel and spring pick and place movement are gathered. These two distributions of data describe an error-free signal sequence from 11 parameter estimations of the delta robot. It can be claimed that if the attained signal sequence from the delta robot while performing either of these tasks does not match these two distributions, there exists anomaly in the data. Thus, there is a problem with delta robot and maintenance is required. The gathered data from the delta robot are 11 time-series from different parameters. Therefore, it is not efficient to use dense neural network in encoding and decoding parts of the AE to learn the underlying distribution of the provided data. To address the aforementioned problems, convolutional layers are used in the structure of the AEs. The architecture of the AEs and its parameter settings are further explained in Section 2.5 and Section 3.4 respectively.

### 2.2. Convolutional Layer

Given the sequential nature of the gathered data, it is decided to use a convolutional layer to capture spatial information and relation in the gathered data. The aforementioned sensor signal sequences are concatenated, and a new dataset is acquired from these separate senor signals. The acquired features from the convolutional layer are calculated as follows. First, several convolutions are performed to compute linear activations as

(3) $$S\left(i,j\right)=\left(X*K\right)\left(i,j\right)=\sum_{m}\sum_{n}X\left(m,n\right)K\left(i-m,j-n\right)$$

where *X* is the concatenated signal sequences and *K* is the two-dimensional kernel. Afterwards, these linear activations are passed through nonlinear activations functions (detector stage). Finally, a pooling function can be applied. It is decided not to use pooling, since it will complicate the neural network in AEs. It can be claimed that the convolutional layer captures all the spatial information required in the new dataset [44,46]. Afterwards, the AEs find a representation for these so-called error-free data points and finally the distribution of these datasets are acquired. Based on these data distributions, a new CI is calculated. Moreover, the aforementioned CI in fact contains information about the distance to data distributions of the error-free working conditions of the delta robot [41]. Thereafter, a probability is assigned to any new signal sequence which describes how probable it is that the delta robot is having a problem given the formerly mentioned CIs.

### 2.3. Delta Robot Dynamics and Parameters

**Figure 1:**
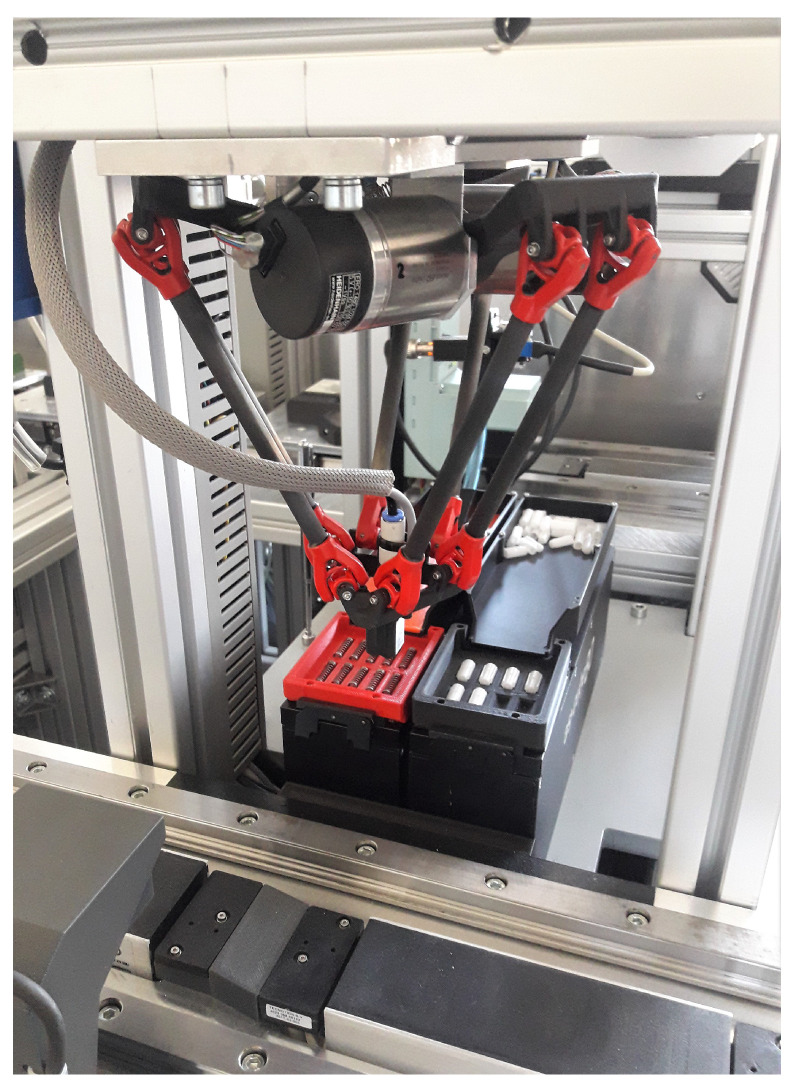
Delta robot as a part of the smart factory.

The studied 3 DoF delta robot is a fully parallel robot with closed loop mechanism. This robot is used for fast and accurate pick and place task. In the conducted study, the delta robot is part of a learning factory, which assembles customized pens according to the requests of the user. The delta robot station is depicted in Figure 1.

Using three parallelograms the moving platform remains at a fixed orientation with respect to the robot base. The movement of the Tool Center Point (TCP) is achieved by the three direct drive motors. The links only transmit force along TCP and are made of carbon fiber tubes. Given the fact that dynamic equation of the studied system is linear in its dynamic parameters, the parameters of the system can be acquired to determine whether the system is operating error-free or not. This can be done using the following equation

**Figure 2:**
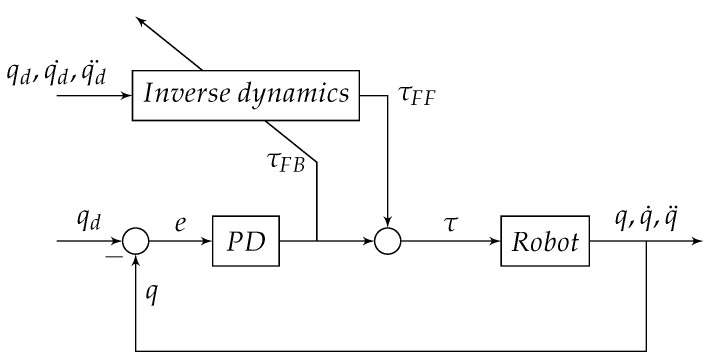
AFFC block diagram. The feed-forward control block contains the parameters the of the delta robot. The parameters will be updated given the output of the feedback controller.

(4) τ=Ψp

where *p* is the dynamic parameter of the system, Ψ is the matrix with dynamic equations of the system and τ is the applied torque. Essentially, the implemented adaptive feed-forward controller (AFFC) in the 3 DoF delta robot, learns the parameters of the system during motion. The block diagram of AFFC can be seen in Figure 2.

Since there are some simplifications in the attained model, to improve the performance of the system, the unmodeled dynamics is compensated with a feedback controller. The control law of the AFFC is calculated as

(5) $$\tau =\Psi \left(q_{d},\dot{q_{d}},\ddot{q_{d}}\right)\;p+\tau_{FB}$$

where $q_{d}$ is the desired joint angle and $\tau_{FB}$ is the torque calculated from the feedback controller, formulated as

(6) $$\tau_{FB}=K_{p}\;e+K_{d}\;\dot{e}$$

The calculated error *e* is the difference between desired and actual joint positions. The terms $K_{p}$ and $K_{d}$ are the proportional and derivative gains of the feedback controller, respectively. While the delta robot is moving, the dynamic parameters of the system (*p*) are learned by the AFFC using the tracking error function. The aforementioned tracking error function is calculated as follows

(7) $$E={\left(\tau -\Psi p\right)}^{2}$$

The implemented learning algorithm is gradient descent and results in the following

(8) $$p_{new}=p_{old}+\alpha \;\Psi^{T}\tau_{FB}$$

where α is a positive definite matrix which determines the learning rates for different parameters. It is worth noting that the aforementioned values only are estimation of the real values [47,48,49]. The aforementioned parameters and the corresponding learning rates in the studied system are shown in Table 1.

### 2.4. Gathering and Preprocessing Data from Delta Robot

To read data from the delta robot and to send commands to it, a local area connection is established with the system and the corresponding HTML page containing all the data in XML format is scraped. Beautiful Soup package from Python is used to scrape the aforementioned page.

After gathering datasets for different tasks of the delta robot, the first step is to normalize the data to remove the effects of scaling in the data. Data normalization can be calculated as follows

(9) $$\tilde{X}=\frac{X-\mu }{\sigma }$$

where μ and σ are the mean and the variance of the gathered data, and $\tilde{X}$ is the normalized data, calculated from the original data points *X* [50]. Another step before feeding the data to the 2d convolutional layer of the proposed method is augmenting another dimension to the data which is similar to different channels of an image.

After preprocessing the gathered data, the next step is to create a training dataset for the proposed method. Given the dynamics of the delta robot in performing different tasks, a window of size 5 data points with stride of 1 is used to create the training dataset. It is worth noting that once the sampling frequency increases, the size of the aforementioned window must increase to capture the same dynamics from the data.

### 2.5. Architecture of the Proposed Method

**Figure 3:**
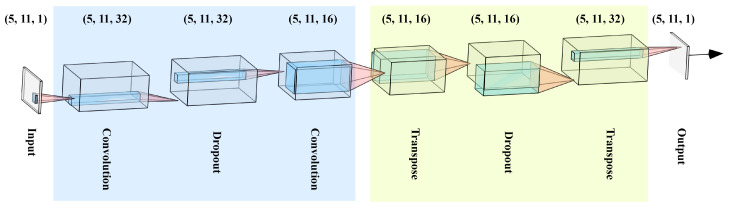
Architecture of the proposed method. Convolutional layers in AE are used to capture information in signal sequences. This architecture tries to replicate the input signal at its output based on the learned data distribution.

The architecture of the proposed method can be seen in Figure 3 [51].

Given the fact that signal sequences from delta robot sensors can be concatenated, it has been decided to use convolutional layers to capture the spatial information in the time-series. Using the AE there is no need to do manual feature engineering and the deep neural network will learn a representation of the distribution of the error-free signal sequences. By replicating the input at the output of the AE, the reconstruction error is used to determine whether a signal sequence is error-free. In the conducted study, the AE is made of the following layers:

- Convolution with 32 filters and kernel size of 4×4
- Dropout
- Convolution with 16 filters and kernel size of 12×12
- Transpose (a.k.a. deconvolution) with 16 filters and kernel size of 12×12
- Dropout
- Transpose with 32 filters and kernel size of 4×4

It is worth noting that the activation function in the convolutional layers is Rectified Linear Unit (ReLU) calculated as follows

(10) $$ReLU\left(x\right)=\left\{\begin{array}{cc} x & \mathrm{if}\;x>0,\\ 0 & \mathrm{otherwise} \end{array}\right.$$

The formerly mentioned activation function can address the vanishing gradient problem, improve the learning speed and lastly, result in positive response of neurons to representations from the features [52,53,54]. It is worth noting that the selected loss function for the neural network is the MSE. More information about the complexity of the architecture and its comparison with other implementations can be found in Section 3.4. In addition, the corresponding code snippet for this part of the implementation can be found in the provided GitHub repository (https://github.com/fathikiavash/Delta_Robot_Predictive_Maintenance, accessed on 12 October 2021).

Based on the dynamics of the system and the sampling rate, the convolution window and the depth of the neural network can be tampered with to come up with the optimal solution for a given system. In the conducted study, two AEs were trained: one for the spring pick and place movement and one for barrel pick and place movement. Based on the reconstruction error the movement can be identified, and it can also be determined whether the delta robot is having a problem and maintenance is required. It is worth noting that the parts of the proposed architecture related to calculating CI and the corresponding HI are implemented using Keras library [55].

### 2.6. GP

In the conducted study, GP is used for performing regression. GPs are non-parametric models which try to assign higher probabilities to functions which have closer values to the available data points or in other words to the functions which fit the dataset better. GPs are in fact an infinite-dimensional multivariate distribution where every selection of random variables has a multivariate Gaussian distribution. Formally, GPs are denoted as $GP(m\left(x\right),k\left(x,x^{\prime }\right))$, where m(x) is the mean function and $k(x,x^{\prime })$ is the covariance function. When *x* and $x^{\prime }$, according to the kernel function, have similar values, the output of the model at these two points will also be similar. Moreover, a collection of random variables {h(x):x∈X} is drawn from GP with mean function *m* and covariance function *k* if the following for $x_{1},x_{2},\ldots ,x_{n}\in X$ holds

(11) $$\left[\begin{array}{c} h(x_{1})\\ \vdots \\ h(x_{n}) \end{array}\right]\;∼\;N\left\{\left[\begin{array}{c} m(x_{1})\\ \vdots \\ m(x_{n}) \end{array}\right],\left[\begin{array}{c} \begin{array}{ccc} k(x_{1},x_{1}) & \cdots & k(x_{1},x_{n})\\ \vdots & \ddots & \vdots \\ k(x_{n},x_{1}) & \cdots & k(x_{n},x_{n}) \end{array} \end{array}\right]\right\}$$

Once a prior is assigned to the parameters of the model *w*, the posterior can be calculated after observing the available data as follows

(12) $$p\left(w|y,X\right)=\frac{p(y|X,w)p(w)}{p(y|X)}$$

where p(y|X,w) is the likelihood, p(w) is the prior and p(y|X) is the marginal likelihood. Thereafter, for a new data point $x^{*}$, the output of the model can be calculated as shown below

(13) $$p\left(f^{*}|x^{*},y,X\right)=\int_{w}p\left(f^{*},x^{*},w\right)p\left(w|y,X\right)dw$$

## 3. Results

In this section, the simulation results are presented for continuous and binary implementation of the proposed method.

### 3.1. Setting up the Model

**Figure 4:**
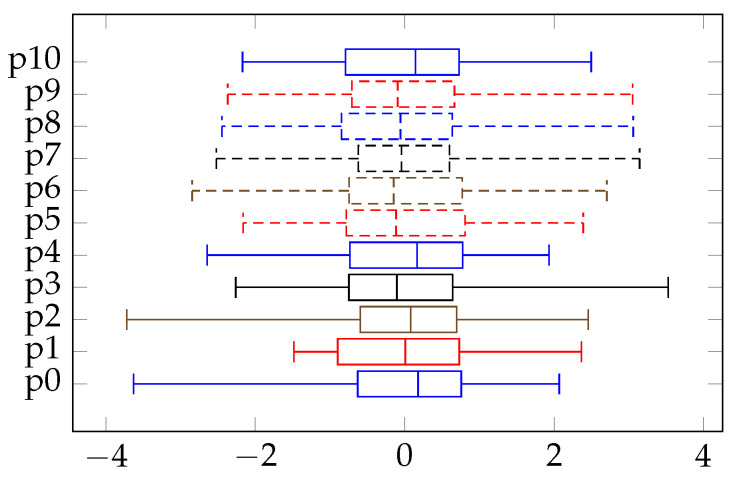
Statistical information of normalized sensor readings of barrel movement.

The delta robot performed spring and barrel pick and place for 900 s, and it was sampled every 100 ms. As an example, the statistical description of the gathered dataset of barrel movement after preprocessing is shown in Figure 4.

**Figure 5:**
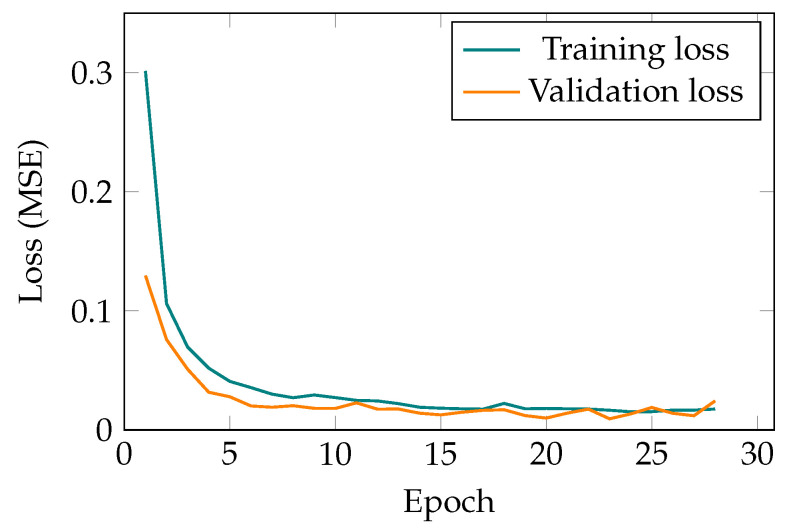
Learning curve of the AE for the spring movement.

The aforementioned datasets are fed to the two AEs for barrel and spring pick and place movement. For demonstration purposes, the learning curves of the deep neural net for the spring movement is shown in Figure 5. As it can be seen in Figure 5, there is no gap between the training and validation final values, which suggests that there is no case of high variance in the training process. Additionally, the MSEs of both training and validation data have converged to a reasonably small value, which denotes that there is no case of high bias either.

**Figure 6:**
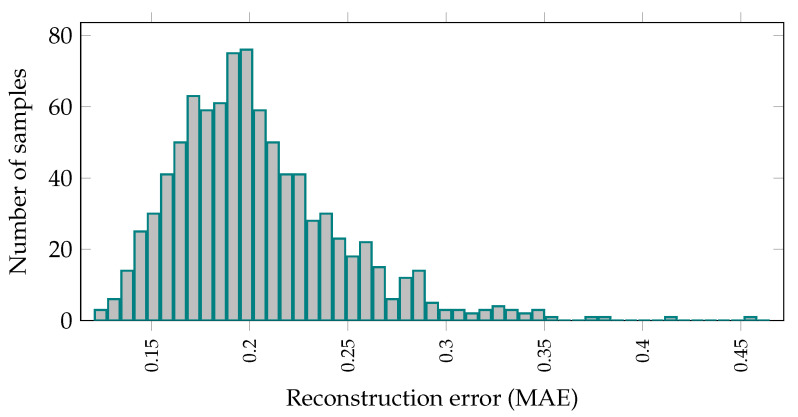
Distribution of reconstruction error given the normalized input data. For the binary classification case, given these data, a threshold can be determined for PdM.

After training AEs, the training dataset is fed to the models and the mean absolute error (MAE) for data points is acquired. Moreover, the maximum MAE is used as the threshold for classifying a signal sequence as anomaly in binary classification case. The simulation results for sample MAE reconstruction errors can be seen in Figure 6.

As pointed out earlier, the maximum reconstruction error is used as a threshold in a binary classification model. In fact, when the reconstruction error is less than this threshold, the given signal sequence will be classified as error-free. It is also possible to come up with a continuous value which describes the probability that a signal sequence is not error-free given the reconstruction error as a CI. As an example, a sigmoid function can be used to map the aforementioned CI to a probability of anomaly which is then used as the HI of the delta robot. The aforementioned sigmoid function is calculated as follows

(14) $$\phi \left(d\right)=\frac{1}{1+e^{-c_{1}\times \left(d-c_{2}\right)}}$$

where *d* is reconstruction error, a.k.a. the CI, which contains information about the deviation from the error-free working condition data distribution of the delta robot. Furthermore, $c_{1}$ is the slope of the sigmoid function and finally, $c_{2}$ determines the point where the sigmoid function is equal to 0.5. Based on the sensitivity of the system, the parameters $c_{1}$ and $c_{2}$ can be chosen. In the conducted study, the threshold for binary classification of anomaly in the barrel and spring movement are 0.46 and 0.30 respectively.

### 3.2. Binary Classification and Predicting Anomaly Probability

**Figure 7:**
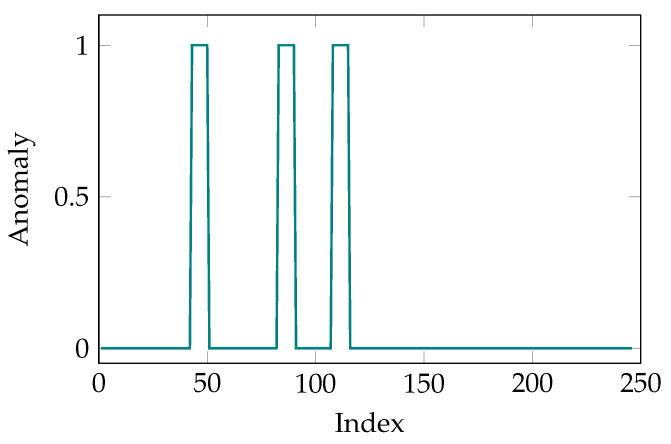
Indices of detected anomalies. Reconstruction errors above a given threshold are depicted as anomaly (1) and otherwise are set to normal (0).

**Figure 8:**
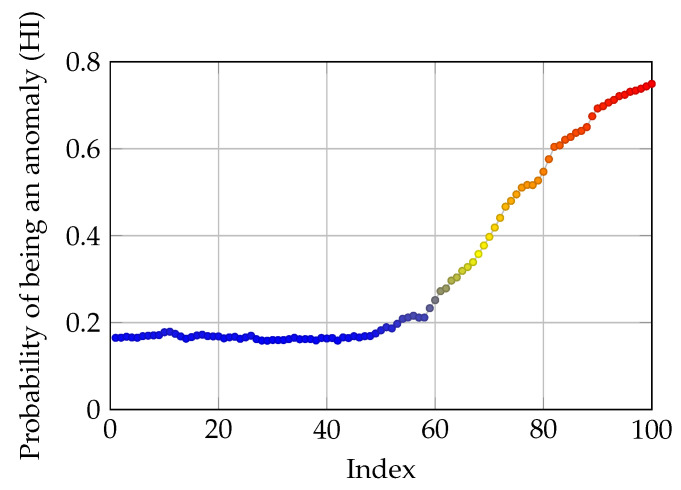
Probability of a signal sequence being an anomaly given the CI.

After training the AEs, a sample dataset is used to test the proposed method. Three sequences of impaired data are created by adding the corresponding signal sequences in the error-free dataset an attenuated noise (maximum amplitude of the noise is 0.1). As an example, an impaired signal sequence of length 4 is created at 45, 85 and 110 indices from the barrel movement dataset. Afterwards, the sample dataset is fed to the AE trained for the barrel movement. It can be seen in Figure 7 that the AE has successfully found the impaired signal sequences. It is worth noting that the binary classification was used in this simulation. Hence, the data points are either denoted as error-free or impaired which are equivalent to 0 and 1, respectively. Moreover, smoother HI values for another anomalous signal sequence from the CI using a sigmoid function with values $c_{1}=1.4$ and $c_{2}=1.6$ are depicted in Figure 8.

**Figure 9:**
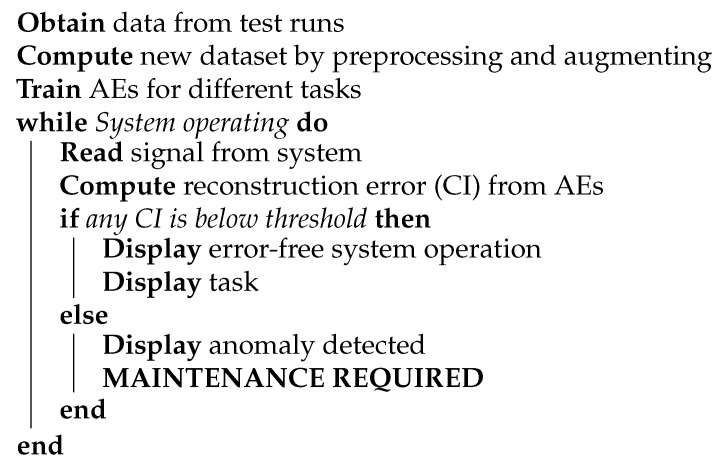
Pseudo-code of the binary classification for erroneous and normal working conditions.

It is worth noting that peak value and also the slope can be tuned to acquire a more case specific output. For example, when the studied system is very susceptible to changes, then a steeper slope and a lower threshold can be used. However, when no domain knowledge about the system is available it is best to use the optimization method introduced in Section 3.5. The pseudo-code of the proposed method can be seen in Figure 9.

### 3.3. Robustness Testing

**Figure 10:**
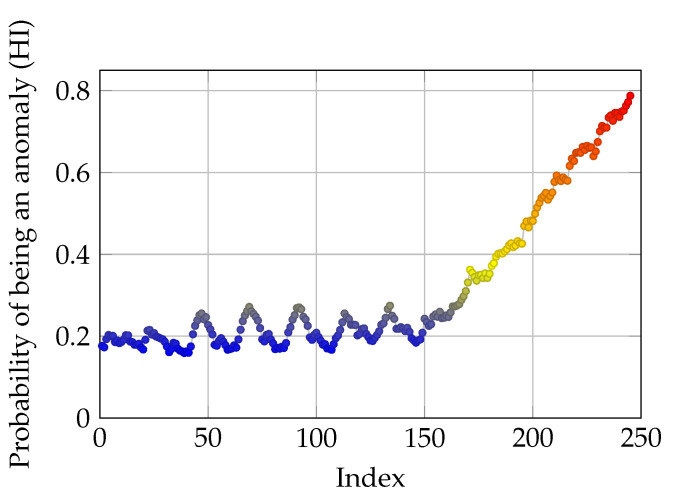
Results of simulation from incremental disturbance. HI values tend to oscillate, due to the partly non-representative training data.

Lastly, to test the robustness of the proposed method, another dataset is acquired from the parameters’ matrix *p* while its values are converging. As described in Section 2.3, the estimated parameters are updated using the tracking error. Therefore, at the early stages when the system has just started working, the values of parameters are not good estimates of the parameters. The aforementioned parameters are converging to the actual value with a predefined learning rate α. The spring AE is trained on this low-quality dataset. Afterwards, another dataset which contains converged values of parameters is tampered with. The length of the test time-series is 245, and from the 150th data point, the corresponding values of the three Coulomb frictions are added with three separate disturbances which increases steadily from 0 until they reach 60% of the maximum value of corresponding friction values. Subsequently, after attaining the new test dataset, this dataset is fed to the spring AE. The simulation results can be seen in Figure 10.

It is worth noting that corresponding $c_{1}$ and $c_{2}$ values are 1.8 and 1.9 respectively. It can be seen that as the friction values diverge from their normal values (error-free), the AE produces CIs which further deviate from the normal values which results in higher HI values. From a PdM point of view, a threshold can be chosen based on the sensitivity of the system and maintenance can be performed based on the calculated HI. Simply, by changing the $c_{1}$ parameter of the sigmoid function, the rate of increase or decrease in the returned probability values can be tuned. On the other hand, by tampering with $c_{2}$, it can be decided which values of attained anomaly probability have to be ignored. In fact, $c_{2}$ is similar to a slack variable which allows divergence from the reference values. Overall, it can be claimed that these two parameters can be fine-tuned according to the studied system and suitable models for PdM can be acquired accordingly. Nonetheless, in the following sections a minimax optimization-based method will also be introduced which provides optimal values for $c_{1}$ and $c_{2}$.

### 3.4. Comparison of the Convolutional, LSTM and Dense AE

As discussed in the introduction, the convolutional layer in the proposed method, significantly enhances the model, namely the captured spatial information in the attained signal sequences, and the acquired features improve the performance of the AE for PdM. The impact of the structure of AE on its performance is studied by training three different AEs:

- AE with dense feed-forward layers: This architecture has the lowest performance and simply is not capable of handling sequential data. Even by increasing the model complexity, this architecture does not show any improvement in the performance. The number of trainable parameters in this case is 475,200. Moreover, the minimum validation loss for this structure is 0.8266.
- AE with LSTM layers: This model has a much better performance than the previous scenario. However, this model cannot outperform the results of the AE with convolutional layers. This simulation results proves that as expected, this structure is capable of handling sequential data, but not as good as the AE with convolutional layers. In this case, the number of trainable parameters is 254,347. It is worth noting that the minimum validation loss for AE with LSTM layers is 0.0637.
- AE with convolutional layers: The best performing among the test models with the fewest parameters. The proposed method has successfully found all the spatial information and acquired features for reconstructing the given signal sequences. The number of parameters in this case is 125,665. Lastly, the minimum validation loss for the chosen AE structure is 0.0281

**Figure 11:**
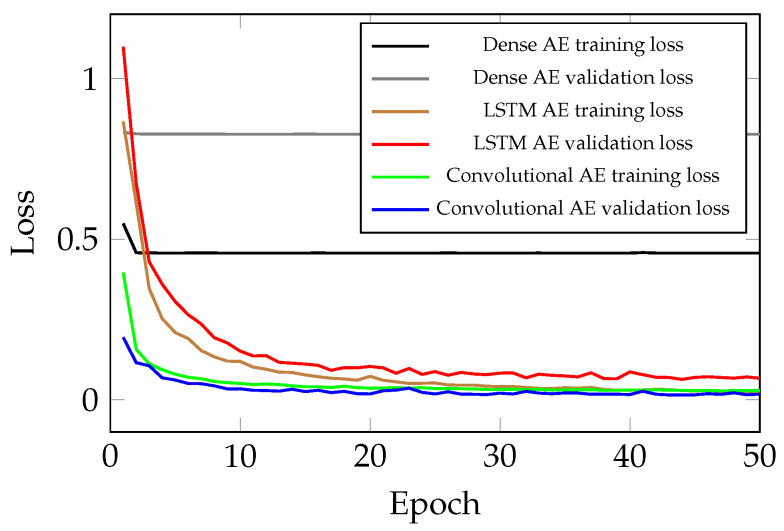
Learning curve of the different AEs for the barrel movement. AE with convolutional layers has the highest generalization power given the validation curves.

The learning curve of the aforementioned models is depicted in Figure 11.

As shown, there is quite a gap between validation loss of the different methods, as an indicator of generalization power of different architectures. As formerly mentioned, the high-dimensional signal sequences from the delta robot can be better processed with an AE which has convolutional layers. In fact, an invaluable source of information is the correlation between different parameter readings, which only can be extracted when convolutional layers are employed. As shown in Section 3.6, after training, some correlations between the parameters of the delta robot are captured, and thus it can be claimed for the studied system the proposed AE structure with convolutional layers is the optimal choice.

### 3.5. Optimization of Sigmoid Function

**Figure 12:**
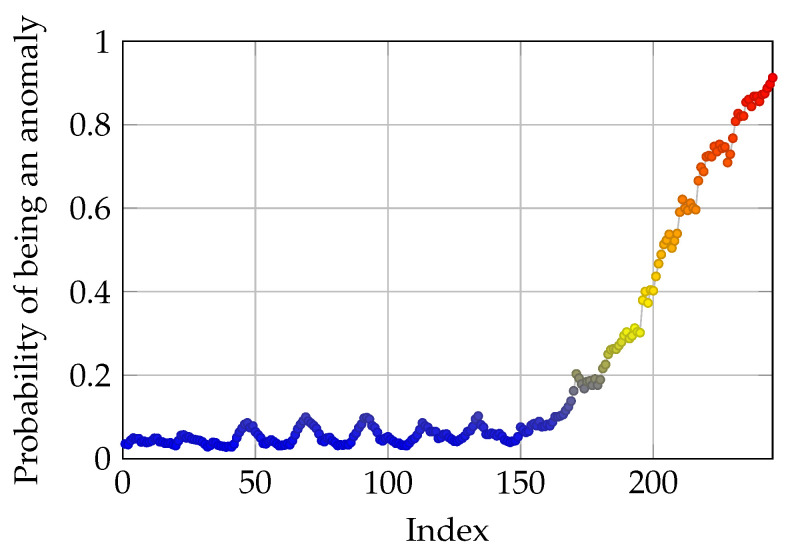
Results of simulation from optimized sigmoid function. Lower oscillation in HI values due to the optimization, which makes the HI time-series more informative.

In this part, to improve the interpretability of the output of the model, the sigmoid function which determines the probability of being an anomaly (HI) is optimized. It is worth noting that this optimization is performed on the test run data which contains no anomaly in different signal sequences. The main idea is to find minimum values of $c_{1}$ and $c_{2}$ while keeping the output sensitive to deviations by maximizing the sigmoid cost function. In other words, a minimax optimization is performed on the sigmoid function, mapping deviation from references, a.k.a. reconstruction errors, to probability of having a problem in the studied system. The optimization problem is formulated as follows

(15) $$\begin{array}{c} C^{*}=\min_{c_{1},c_{2}}\max_{i}f_{i},\;i\in 1,2\\ f_{1}=\sum_{d}^{D}\frac{1}{1+e^{-c_{1}\left(d-c_{2}\right)}}\\ f_{2}=std\left(\frac{1}{1+e^{-c_{1}\left(D-c_{2}\right)}}\right) \end{array}$$

where $C^{*}$ is the optimal parameters $c_{1}$ and $c_{2}$, given the aforementioned optimization problem and initial values of $c_{1}$ and $c_{2}$. Additionally, *D* is the vector of calculated *d* for all the data points in the test run signal sequences. Furthermore, std is the standard deviation of applied sigmoid function on *D*, with $c_{1}$ and $c_{2}$ parameters. The maximization part of the above optimization is for ensuring that the output of the optimization problem is not a flat signal of zero with large values of $c_{1}$ and $c_{2}$. Moreover, the minimization forces the $c_{1}$ and $c_{2}$ parameters to converge to values which make the sigmoid function have lower bias and a gentle slope. The results of optimization is depicted in Figure 12.

A ramp disturbance is added to the available spring movement from the 150th data point. The data used in this part of the study are the same as the previous section, as shown in Figure 10, where the robustness of the model was tested. As it can be seen in Figure 12, the optimized probability function, tries to keep the probability of anomaly for the first 150 data points zero, and just as the disturbance and deviation is added to the original data, the probability of anomaly increases steadily. By requiring more variance in the optimization problem, a more informative mapping from deviation to anomaly probability is acquired. Moreover, $c_{1}$ and $c_{2}$ values are 3.56 and 1.96 respectively.

### 3.6. Fault Localization

**Figure 13:**
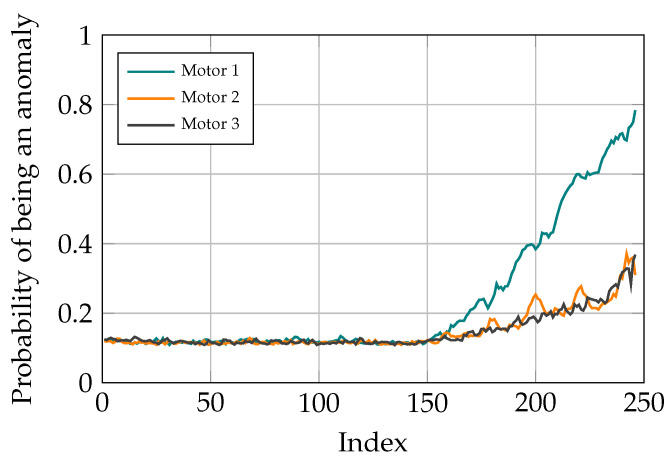
Simulation results with distortion added to motor 1.

**Figure 14:**
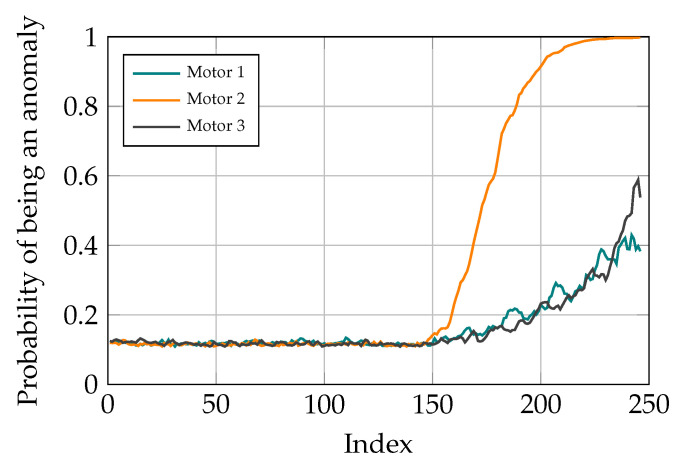
Simulation results with distortion added to motor 2.

**Figure 15:**
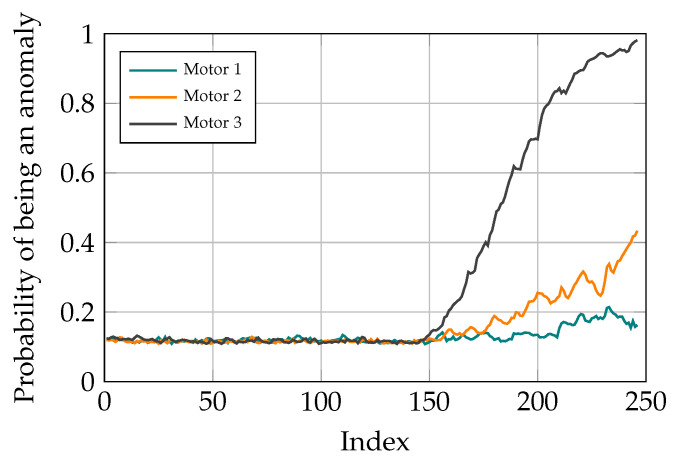
Simulation results with distortion added to motor 3.

After finding out the fact that there is anomaly in the studied system and therefore, a need for maintenance, the next task is fault localization. For doing so, after classifying the current task through the signal sequence distribution with AEs, the contribution of different reconstruction errors is analyzed to determine where the problem originates from. In the conducted study, there are three motors for moving the TCP of the delta robot and the goal is to find out which of these motors is not operating well. Different scenarios are studied to show the capability of the proposed method for fault localization. To do so, different ramp disturbances are added to Coulomb and Viscose friction of the motors in the spring movement dataset from the 150th data point. The final value of these disturbances is 60% of the maximum value of the friction values. The CI for different motors is determined by the sum of reconstruction errors in Coulomb and Viscose friction passed through the sigmoid function. As it can be seen in Figure 13, Figure 14 and Figure 15, the model is able to pinpoint where the problem derives from. Noticeably, the anomaly probability increases steadily from the 150th data point for all the scenarios.

**Figure 16:**
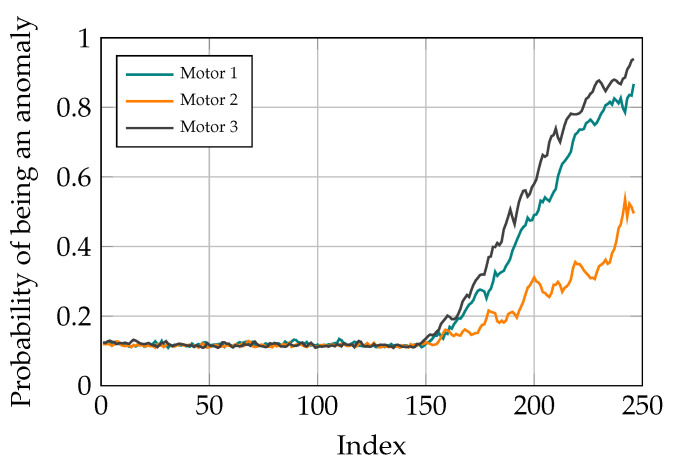
Simulation results with distortion added to motors 1 and 3.

**Figure 17:**
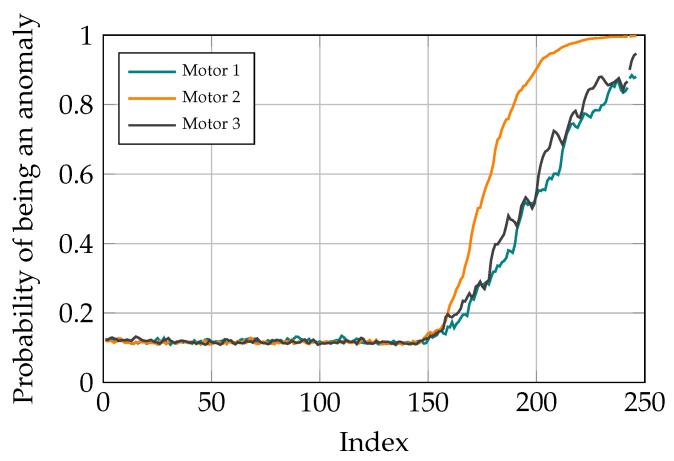
Simulation results with distortion added to motors 1, 2 and 3.

Similarly, when two or more friction values are manipulated, the model can determine the fault in the system. Moreover, the anomaly probabilities higher than a predetermined threshold depict which motors are having problem and need maintenance. Two examples are shown in Figure 16 and Figure 17, where the friction values of the first and third motor and all the motors are manipulated, respectively.

The probability of being an anomaly given the CI, is calculated similar to the previous sections, with the $c_{1}$ and $c_{2}$ values of 1.8 and 1.3 respectively. Given the dynamic of parallel robots, when there is a disturbance in the friction values of a motors, it can be seen that all the reconstruction errors in different motors increase. This shows that the trained model has learned the correlation between different parameter values and thus, is not purely an identity function which tries to replicate the input at its output. This characteristic of the model can significantly help increase the interpretability of the trained model.

### 3.7. RUL

**Figure 18:**
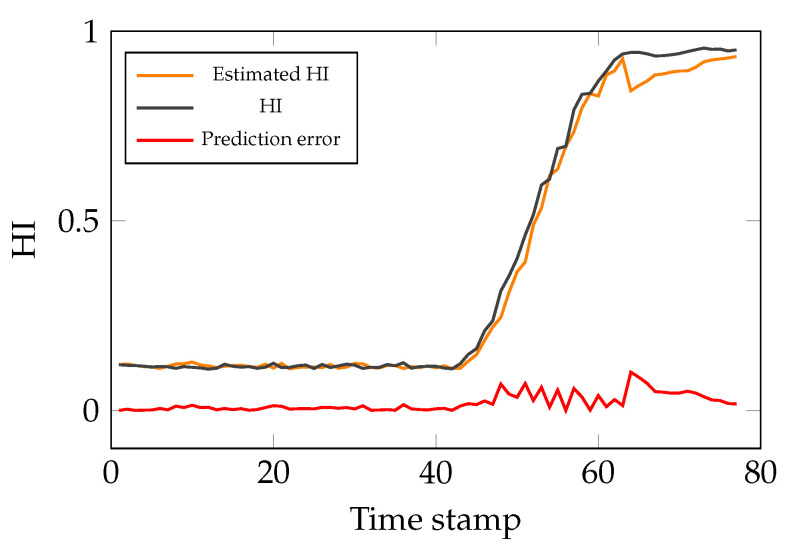
Simulation results for HI estimation using the degradation model.

**Figure 19:**
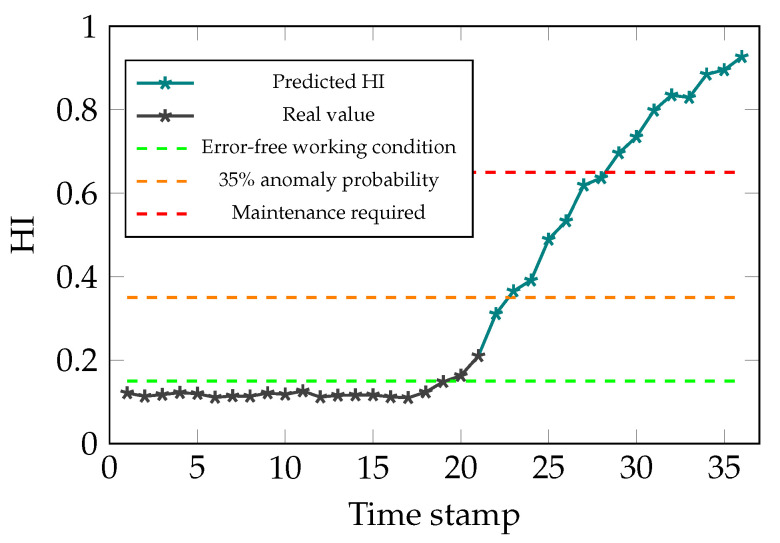
Simulation results for RUL estimation.

After acquiring a HI which determines the probability of having an anomaly given the CI, the next and last step is to calculate RUL. In fact, using the AEs, raw parameter readings from the delta robot are converted to new CIs. Afterwards, by calculating the deviation from reference values for the aforementioned CIs, the probability of having an anomaly given CI is calculated as the HI of the studied system. Moreover, in the last step, this value is predicted to estimate RUL of the studied system. Additionally, given the fact that no R2F data from the system is available, threshold values for the anomaly probability values can be used to predict RUL. In fact, by having the aforementioned thresholds, RUL is calculated as the difference between the current time stamp and the time stamp when the HI is higher than the threshold. Furthermore, for calculating the HI in future time stamps, a GP for modelling degradation is trained to predict the anomaly probability values. The input for the GP is 20 past HI data points and afterwards, 15 future HI data points are predicted. It is worth noting that the kernel is designed only once, and the GP is (re)trained once the new data points are available. Moreover, for acquiring better results and reducing calculations, it is recommended to use an appropriate stride value for retraining the GP. In the conducted study, the CIs are used to train the GP with a stride of 6. In this case, in every step the GP required on average 0.02936 s to be retrained. The results of the conducted study for the degradation model and RUL calculation can be seen in Figure 18 and Figure 19.

As shown in Figure 18, the GP can predict the future HI values of the system. It should be noted that the aforementioned figure contains plots of the HI of the system though time and the predicted value from the degradation model. The GP regressor has a MSE of 0.00092. Moreover, the explained variance score of the GP is 99.41% which is calculated as follows:

(16) $$explained\;variance\left(y,\hat{y}\right)=1-\frac{Var\{y-\hat{y}\}}{Var\{y\}}$$

where *y* is the HI of the system, $\hat{y}$ is the predicted value using the GP and Var is the variance of the formerly mentioned values. As it can been seen in Figure 19, the probability of having an anomaly, has steadily increased from the 18th time stamp. Additionally, the predictions from the degradation model suggest that in 8 steps the system will reach a critical condition and therefore, maintenance is required prior to the 29th time stamp. Thus, it can be claimed that the RUL of the system is 7 time stamps given the assigned threshold, which is anomaly probability value of 65%, and the applied degradation model. It is worth noting that given the stride value of the GP, one time stamp is equivalent to 6 sampling times for reading the parameters from the system. Moreover, the used kernel for the GP is made of the following parts:

1. Exp-Sine-Squared kernel
  (17) $$k\left(x,x^{\prime }\right)=exp\left(-\frac{2sin^{2}\left(\pi d\left(x,x^{\prime }\right)/p\right)}{l^{2}}\right)$$
2. Radial basis function
  (18) $$k\left(x,x^{\prime }\right)=exp\left(-\frac{d{\left(x,x^{\prime }\right)}^{2}}{2l^{2}}\right)$$
3. Matern
  (19) $$k\left(x,x^{\prime }\right)=\frac{2^{1-\nu }}{\Gamma (\nu )}{\left(\sqrt{2\nu }\frac{d(x,x^{\prime })}{l}\right)}^{\nu }K_{\nu }\left(\sqrt{2\nu }\frac{d(x,x^{\prime })}{l}\right)$$
4. Constant kernel
  (20) $$k\left(x,x^{\prime }\right)=constant\;value\;\forall x,x^{\prime }\in X$$
  where $d(x,x^{\prime })$ is the Euclidean distance between two data points *x* and $x^{\prime }$. Additionally, *p* is the periodicity parameter, *l* is the length scale, ν controls the smoothness of the kernel, $K_{\nu }$ is a modified Bessel function and finally, Γ is the gamma function. It is worth noting that the implementation of the degradation model is done using the scikit−learn library [56].

## 4. Discussion

In this paper, a semi-supervised hybrid deep neuron network structure was introduced for finding anomaly in signal sequences acquired from sensor readings of a delta robot for PdM. The delta robot performed pick and place of barrels and springs and was part of a smart factory for assembling customized pens. The main notion of the proposed method is to find anomaly in the data sequence and assign an anomaly probability accordingly. The proposed structure did not require any R2F signal sequence samples of the system for different tasks. The only requisite of this method was to have enough data from different tasks for the AEs to find the underlying distribution of the error-free signal sequences. This characteristic is vital when there are not enough samples of failure available due to high costs or the danger it causes. In fact, as the system becomes more complicated, it is not possible to claim that all the different signal sequences leading to different failures are captured and stored. Therefore, it is better to predict error-free working conditions of the system and afterwards, flag the signal sequences which do not correspond to the error-free data sequences as anomaly. In every step, the AEs were given the acquired signals from sensor readings and, they tried to replicate these signals at their output. In addition, in the binary classification case, if any of these AEs had a reconstruction error less than the previously assigned threshold, the provided signal sequence would be classified as error-free. It was also possible to determine the task, given the fact that only the AE, trained on that specific task, would have a reconstruction error less than the threshold. Additionally, the threshold-based decision making is where the current literature employs the acquired reconstruction errors. Nonetheless, our method goes beyond just calculating deviations. In fact, the reconstruction error was used as a new CI for the studied system, which was afterwards used to calculate the HI of the system. Furthermore, the simulation results showed that the trained AEs had successfully learned the distribution of spring and barrel pick and place movement. Moreover, when the error-free signal sequence was disturbed with noise, the AEs were able to find the indices of data which were tampered with. Additionally, using a sigmoid function and assigning appropriate parameters regarding the studied system, a continuous value, as an anomaly probability value, defined as the HI of the system, could be acquired. The HI then could be used to determine whether the system required maintenance given the thresholds. In fact, the acquired HI is more informative than the raw reconstruction error values, and it can be used to determine the current state of the system, which is namely another contribution of the conducted study. Furthermore, the aforementioned sigmoid function could be optimized by solving a minimax problem, and the acquired parameters were in fact the saddle point of this optimization problem. Additionally, the aforementioned approach was effective when not enough domain knowledge about the system was available. It is worth noting that this function, could also be manually tuned based on the sensitivity of the studied system and the needs of the user. In addition, it was also shown that the most effective architecture based on AEs for performing PdM was a combination of convolutional layers for extracting features from the available high-dimensional raw data and afterwards, finding the signal sequence of different tasks accordingly. Thereafter, the trained deep neural networks were used for fault localization. The results of this part of the study showed that the trained model was capable of pinpointing where the problem originated from, assuming that distortions originated from the internal parts of the system. Moreover, when two or more motors were misbehaving in the delta robot, the proposed method was able to distinguish which motors were not working well. Additionally, the anomaly signal sequences in fault localization depicted the fact that the trained model has learned some correlation between signal sequences. Moreover, the results suggested that the trained AEs were not merely an identity function trying to replicate their input at the output. Lastly, the recently calculated HIs were used to predict the RUL, using a GP regressor as a degradation model. In fact, the aforementioned GP was retrained after acquiring new parameter readings from the delta robot. Moreover, once new parameter readings were available, the corresponding CIs were calculated and finally the HI of the system given the CIs were acquired. Thereafter, the new sequence of HIs were sent to the degradation model to be retrained. Afterwards, the degradation model predicted the future HI values of the system. As an example, in the conducted studies 15 future time stamps were predicted by having the last 20 HI values of the system. Subsequently, it was possible to use a threshold data to assign a time in future for performing PdM given the predictions of the degradation model. In addition, the trained degradation model was able to capture the trends in the HI value and the acquired explained variance score was 99.41%. It is vital to remember that no R2F data were available in the conducted study and thus the formerly explained approach was used for calculating HI and RUL of the system. It is worth noting that this approach is most likely to work on systems similar to the delta robot introduced in this paper. However, for more complicated systems, which show highly nonlinear behavior in their degradation value patterns, this model is most likely to fail. In such cases, it is important to have enough R2F data to compensate for the complexity of the degradation model of such systems. In addition, the proposed architecture is superior to analytical models given the following reasons. Using AEs, a movement dependent anomaly detection model can be trained. In addition, the proposed model relies merely on the read data from the system and thus can be fine-tuned given new parameter readings. Moreover, the stochastic nature of the architecture helps to model the uncertainties in the real system and the acquired data. In fact, by employing soft computing, it is possible to account for both small deviations from the acceptable parameter readings and deviations that will lead to an anomaly. Furthermore, unlike the proposed method, for an accurate estimation of RUL, a very precise analytical model is required, which can be hard to obtain as the system becomes more complex. However, it can be claimed that by employing an analytical model along with an AE architecture, a relatively simpler analytical model can be used for estimating the RUL. Moreover, using an analytical model, a considerable amount of domain knowledge is contained in the analytical model. Thereafter, the AE can be used to determent deviations from the error-free parameter sequences given the available domain knowledge from the analytical model. In short, given a complex system, a combination of an analytical model and the AE architecture can determine the RUL of the system. However, for a simpler system, similar to the studied delta robot, a combination of AE and GP can determine the RUL. Moreover, as the HI deteriorates, the impact of the precision of the analytical model decreases, due to the fact that at extreme the HI is not far from point of failure. Lastly, in the case of minor changes in the physical system, the proposed method only must be retrained given the new data acquired from the system and no further updates in the remaining parts of the proposed model is required.

## 5. Conclusions

The conducted study addresses the PdM problem without the access to R2F data, as is the case with numerous industrial applications [22]. Moreover, given the lack of availability of R2F data, it was required to develop multiple computational layers to solve the PdM problem in a semi-supervised manner. In fact, the proposed model was made of two parts: the first part was responsible for calculating the deviations from error-free signal sequence distributions, a.k.a. CIs, and thereafter calculating the estimated HI of the system. The second part; however, was responsible for predicting the future HI values of the system, from which the RUL of the system could be inferred. Moreover, the first part of the architecture was trained in a semi-supervised manner, due to the AE capabilities for finding structures in parameter readings of the robot. Compared to the state-of-the-art, to the best of our knowledge, no other method has tried to solve the PdM problem without the access to R2F data in an optimized way. As shown in the conducted experiments, the proposed architecture, could find deviations from error-free signal sequences exquisitely. Thereafter, it was shown that based on the read data from the robot, a minimax optimization problem could be solved, which determined the optimal probability function for mapping CI to HI values. Nonetheless, the sigmoid function, used as the probability function, can also be hand tuned when domain knowledge regarding the system is available. The degradation model, which was a GP regressor, could predict the future HI values given its assigned kernels. In fact, different kernels of the GP tried to model different characteristics of the HI sequence. Additionally, it was shown that given an error in the friction values of the delta robot, it was also possible to determine where the problem originated form, as the proposed architecture had also found some correlations between different parameter readings of the system. The proposed method is vital for scenarios, where gathering R2F data can be dangerous, time consuming and/or expensive. The complexity of the studied system can to some extent affect the performance of the proposed architecture. As one of the future works of the conducted study, system which are more complex or have higher degrees of nonlinearity could be studied and some improvements in the computational layers could be made. Moreover, another aspect of the current work which could be explored, is the end-to-end development and optimization of the aforementioned layers for a more united architecture for PdM. Lastly, the degradation model could be further improved in such a way that the trends learned by the kernels could also be manipulated by an expert, e.g., a system’s engineer, and hence result in a more domain specific implementation of the model.

## Figures and Tables

**Table 1:** Parameters and the corresponding learning rates.

Parameter	Learning Rate
Motor inertia [gm${}^{2}$]	0.0005
Gravity [mN]	0.08
Mass [g]	0.09
Spring offset [mN m]	0.07
Coulomb friction 0 [mNm]	0.1
Coulomb friction 1 [mNm]	0.1
Coulomb friction 2 [mNm]	0.1
Spring constant [mNm/rad]	0.2
Viscose friction 0 [mNm s/rad]	0.01
Viscose friction 1 [mNm s/rad]	0.01
Viscose friction 2 [mNm s/rad]	0.01

## Data Availability

The data presented in this study are openly available in Mendeley Data at doi:10.17632/fddp3dvvzr.1.

## Abbreviations

The following abbreviations are used in this manuscript:

PdM	Predictive maintenance
R2F	Run-to-failure
CI	Condition indicator
HI	Health index
RUL	Remaining useful lifetime
AE	Autoencoder
GP	Gaussian process
LSTM	Long short-term memory
MSE	Mean squared error
AFFC	Adaptive feed-forward controller
MAE	Mean absolute error

## References

[1] Mobley R.K. (2002). An Introduction to Predictive Maintenance.

[2] Schmidt B., Wang L. (2018). Cloud-enhanced predictive maintenance. Int. J. Adv. Manuf. Technol..

[3] Krupitzer C., Wagenhals T., Züfle M., Lesch V., Schäfer D., Mozaffarin A., Edinger J., Becker C., Kounev S. (2020). A survey on predictive maintenance for industry 4.0. arXiv.

[4] Luo W., Hu T., Ye Y., Zhang C., Wei Y. (2020). A hybrid predictive maintenance approach for CNC machine tool driven by Digital Twin. Robot. Comput. Integr. Manuf..

[5] Butler K.L. An expert system based framework for an incipient failure detection and predictive maintenance system. Proceedings of the International Conference on Intelligent System Application to Power Systems.

[6] Shimada J., Sakajo S. A statistical approach to reduce failure facilities based on predictive maintenance. Proceedings of the 2016 International Joint Conference on Neural Networks (IJCNN).

[7] Li C., Zhang Y., Xu M. (2012). Reliability-based maintenance optimization under imperfect predictive maintenance. Chin. J. Mech. Eng..

[8] Ortiz J., Carrasco R.A. (2016). Model-based fault detection and diagnosis in ALMA subsystems. Observatory Operations: Strategies, Processes, and Systems VI.

[9] Lei Y., Li N., Gontarz S., Lin J., Radkowski S., Dybala J. (2016). A model-based method for remaining useful life prediction of machinery. IEEE Trans. Reliab..

[10] Canizo M., Onieva E., Conde A., Charramendieta S., Trujillo S. Real-time predictive maintenance for wind turbines using Big Data frameworks. Proceedings of the 2017 IEEE international conference on prognostics and health management (ICPHM).

[11] Wang J., Fu P., Zhang L., Gao R.X., Zhao R. (2019). Multilevel information fusion for induction motor fault diagnosis. IEEE/ASME Trans. Mechatron..

[12] Levitt J. (2003). Complete Guide to Preventive and Predictive Maintenance.

[13] Goyal D., Pabla B.S. (2015). Condition based maintenance of machine tools—A review. IRP J. Manuf. Sci. Technol..

[14] Lughofer E., Sayed-Mouchaweh M. (2019). Predictive Maintenance in Dynamic Systems: Advanced Methods, Decision Support Tools and Real-World Applications.

[15] Cheng J.C., Chen W., Chen K., Wang Q. (2020). Data-driven predictive maintenance planning framework for MEP components based on BIM and IoT using machine learning algorithms. Autom. Constr..

[16] Baptista M., Sankararaman S., de Medeiros I.P., Nascimento C., Prendinger H., Henriques E.M. (2018). Forecasting fault events for predictive maintenance using data-driven techniques and ARMA modeling. Comput. Ind. Eng..

[17] Yu W., Kim I.Y., Mechefske C. (2020). An improved similarity-based prognostic algorithm for RUL estimation using an RNN autoencoder scheme. Reliab. Eng. Syst. Saf..

[18] Da Xu L., He W., Li S. (2014). Internet of things in industries: A survey. IEEE Trans. Ind. Inform..

[19] Li X., Li D., Wan J., Vasilakos A.V., Lai C.F., Wang S. (2017). A review of industrial wireless networks in the context of industry 4.0. Wirel. Netw..

[20] Yan J., Meng Y., Lu L., Li L. (2017). Industrial Big Data in an Industry 4.0 Environment: Challenges, Schemes, and Applications for Predictive Maintenance. IEEE Access.

[21] Kiangala K.S., Wang Z. (2018). Initiating predictive maintenance for a conveyor motor in a bottling plant using industry 4.0 concepts. Int. J. Adv. Manuf. Technol..

[22] Cattaneo L., Macchi M. (2019). A Digital Twin Proof of Concept to Support Machine Prognostics with Low Availability of Run-To-Failure Data. IFAC-PapersOnLine.

[23] Rengasamy D., Jafari M., Rothwell B., Chen X., Figueredo G.P. (2020). Deep Learning with Dynamically Weighted Loss Function for Sensor-Based Prognostics and Health Management. Sensors.

[24] Rengasamy D., Morvan H.P., Figueredo G.P. Deep learning approaches to aircraft maintenance, repair and overhaul: A review. Proceedings of the 2018 21st International Conference on Intelligent Transportation Systems (ITSC).

[25] Jogin M., Madhulika M.S., Divya G.D., Meghana R.K., Apoorva S. Feature extraction using Convolution Neural Networks (CNN) and Deep Learning. Proceedings of the 2018 3rd IEEE International Conference on Recent Trends in Electronics, Information & Communication Technology (RTEICT).

[26] Reddy K.K., Sarkar S., Venugopalan V., Giering M. Anomaly detection and fault disambiguation in large flight data: A multi-modal deep auto-encoder approach. Proceedings of the Annual Conference of the Prognostics and Health Management Society.

[27] Sarkar S., Reddy K.K., Giering M. Deep learning for structural health monitoring: A damage characterization application. Proceedings of the Annual Conference of the Prognostics and Health Management Society.

[28] Yuan M., Wu Y., Lin L. Fault diagnosis and remaining useful life estimation of aero engine using LSTM neural network. Proceedings of the 2016 IEEE International Conference on Aircraft Utility Systems (AUS).

[29] Susto G.A., Schirru A., Pampuri S., McLoone S., Beghi A. (2014). Machine learning for predictive maintenance: A multiple classifier approach. IEEE Trans. Ind. Inform..

[30] Kiangala K.S., Wang Z. (2020). An Effective Predictive Maintenance Framework for Conveyor Motors Using Dual Time-Series Imaging and Convolutional Neural Network in an Industry 4.0 Environment. IEEE Access.

[31] Fan C., Sun Y., Zhao Y., Song M., Wang J. (2019). Deep learning-based feature engineering methods for improved building energy prediction. Appl. Energy.

[32] Rutagarama M. (2019). Deep Learning for Predictive Maintenance in Impoundment Hydropower Plants. Ph.D. Thesis.

[33] Sutskever I., Vinyals O., Le Q.V. (2014). Sequence to sequence learning with neural networks. Advances in Neural Information Processing Systems.

[34] Que Z., Liu Y., Guo C., Niu X., Zhu Y., Luk W. Real-time Anomaly Detection for Flight Testing using AutoEncoder and LSTM. Proceedings of the 2019 International Conference on Field-Programmable Technology (ICFPT).

[35] Hundman K., Constantinou V., Laporte C., Colwell I., Soderstrom T. Detecting spacecraft anomalies using lstms and nonparametric dynamic thresholding. Proceedings of the 24th ACM SIGKDD International Conference on Knowledge Discovery & Data Mining.

[36] Yang J., Nguyen M.N., San P.P., Li X.L., Krishnaswamy S. Deep convolutional neural networks on multichannel time series for human activity recognition. Proceedings of the Twenty-Fourth International Joint Conference on Artificial Intelligence.

[37] Mishra K.M., Krogerus T.R., Huhtala K.J. Fault detection of elevator systems using deep autoencoder feature extraction. Proceedings of the 2019 13th International Conference on Research Challenges in Information Science (RCIS).

[38] Sakurada M., Yairi T. Anomaly detection using autoencoders with nonlinear dimensionality reduction. Proceedings of the MLSDA 2014 2nd Workshop on Machine Learning for Sensory Data Analysis.

[39] Essien A., Giannetti C. (2020). A deep learning model for smart manufacturing using convolutional LSTM neural network autoencoders. IEEE Trans. Ind. Inform..

[40] Wu J., Zhao Z., Sun C., Yan R., Chen X. (2020). Fault-attention generative probabilistic adversarial autoencoder for machine anomaly detection. IEEE Trans. Ind. Inform..

[41] Chandola V., Banerjee A., Kumar V. (2009). Anomaly detection: A survey. ACM Comput. Surv. (CSUR).

[42] Yan J., Koc M., Lee J. (2004). A prognostic algorithm for machine performance assessment and its application. Prod. Plan. Control.

[43] Charu C.A. (2018). Neural Networks and Deep Learning: A Textbook.

[44] Goodfellow I., Bengio Y., Courville A. (2016). Deep Learning.

[45] Hinton G.E., Salakhutdinov R.R. (2006). Reducing the dimensionality of data with neural networks. Sci. Am. Assoc. Adv. Sci..

[46] Brownlee J. (2018). Deep Learning for Time Series Forecasting: Predict the Future with MLPs, CNNs and LSTMs in Python.

[47] Arrazate R.T. (2017). Development of a URDF File for Simulation and Programming of a Delta Robot Using ROS. Master’s Thesis.

[48] Honegger M., Codourey A., Burdet E. Adaptive control of the hexaglide, a 6 dof parallel manipulator. Proceedings of the International Conference on Robotics and Automation.

[49] Codourey A., Burdet E. A body-oriented method for finding a linear form of the dynamic equation of fully parallel robots. Proceedings of the International Conference on Robotics and Automation.

[50] Raschka S., Mirjalili V. (2017). Python Machine Learning.

[51] LeNail A. (2019). Nn-svg: Publication-ready neural network architecture schematics. J. Open Source Softw..

[52] Ide H., Kurita T. Improvement of learning for CNN with ReLU activation by sparse regularization. Proceedings of the 2017 International Joint Conference on Neural Networks (IJCNN).

[53] Nair V., Hinton G.E. Rectified linear units improve restricted boltzmann machines. Proceedings of the ICML 2010.

[54] Glorot X., Bordes A., Bengio Y. Deep sparse rectifier neural networks. Proceedings of the Fourteenth International Conference on Artificial Intelligence and Statistics.

[55] François C. (2015). Keras. https://keras.io.

[56] Pedregosa F., Varoquaux G., Gramfort A., Michel V., Thirion B., Grisel O., Blondel M., Prettenhofer P., Weiss R., Dubourg V. (2011). Scikit-learn: Machine learning in Python. J. Mach. Learn. Res..

